# The Tardive Dyskinesia Impact Scale (TDIS), a novel patient-reported outcome measure in tardive dyskinesia: development and psychometric validation

**DOI:** 10.1186/s41687-023-00679-4

**Published:** 2024-01-04

**Authors:** Robert H. Farber, Donald E. Stull, Brooke Witherspoon, Christopher J. Evans, Charles Yonan, Morgan Bron, Rahul Dhanda, Eric Jen, Christopher O.’ Brien

**Affiliations:** 1 Neurocrine Biosciences Inc., 12780 El Camino Real, San Diego, CA 92130 USA; 2 IQVIA, Durham, NC USA; 3 Lumanity, Boston, MA USA

**Keywords:** Tardive dyskinesia, Validation, Patient-reported outcomes, Psychometrics

## Abstract

**Background:**

Tardive dyskinesia (TD), a movement disorder in which patients experience abnormal involuntary movements, can have profound negative impacts on physical, cognitive, and psychosocial functioning. The Abnormal Involuntary Movement Scale (AIMS), a clinician-rated outcome, is considered the gold standard for evaluating treatment efficacy in TD clinical trials. However, it provides little information about the impacts of uncontrolled movements from a patient perspective and can be cumbersome to administer in clinical settings. The Tardive Dyskinesia Impact Scale (TDIS) was developed as a patient-reported outcome measure to fulfill the need for a disease-specific impact assessment in TD. The objective of the present study was to develop and evaluate the psychometric properties of the TDIS to determine whether it is fit-for-purpose to measure TD impact.

**Methods:**

Data from qualitative studies and phase 3 trials of a VMAT2 inhibitor for the treatment of TD (KINECT3 and KINECT4) were used to determine the psychometric properties of the TDIS. Qualitative research included concept elicitation and cognitive debriefing interviews with TD patients and their caregivers in order to assess how well the TDIS captured key domains of TD impact. Quantitative analyses to examine the psychometric properties of the TDIS included assessing construct validity (factor structure, known groups, and predictive validity) and responsiveness to change.

**Results:**

Qualitative results showed that the TDIS captures the key TD impacts reported by patients and caregivers and that the TDIS was interpreted as intended and relevant to patients’ experiences. Quantitative results found evidence of 2 underlying domains of the TDIS: physical and socioemotional (Comparative Fit Index > 0.9). Known groups and predictive validity indicated that, compared with the AIMS, the TDIS captures unique content (correlation between AIMS and TDIS = 0.2–0.28). The TDIS showed responsiveness to change in treatment, with TDIS scores improving over 48 weeks in the 2 phase 3 trials.

**Conclusions:**

The TDIS captures relevant information about the impact of TD and is easily administered in a clinician’s office or patient’s home. It may be used longitudinally to show changes in TD burden over time. The TDIS complements the AIMS; using these assessments together provides a more holistic assessment of TD.

## Abstract (plain language summary)

Tardive dyskinesia is a condition where people have uncontrollable movements because of taking certain medications for a long time. It is still poorly understood how these uncontrollable movements affect a person’s everyday activities. We created a questionnaire called the Tardive Dyskinesia Impact Scale (TDIS). The TDIS is a questionnaire where people with tardive dyskinesia rate how their symptoms affect daily activities such as speaking and walking. People can also rate how the uncontrollable movements make them feel. We used specific tests called psychometric tests to see if the TDIS measures the correct information and if the information is reliable. Findings from this study show that the TDIS is a good way to measure how a person’s uncontrollable movements affect everyday activities. The results also show that when people take medicine to help with their symptoms, their TDIS scores are better. When patients stopped taking the medicine, their symptoms were worse, and their TDIS score was worse. The TDIS can help people explain how their uncontrollable movements affect their daily life. This can then help their doctors understand the person’s condition better.

## Background

Tardive dyskinesia (TD) is an involuntary, hyperkinetic, and potentially disabling movement disorder in which patients experience abnormal involuntary movements. These movements are the result of long-term exposure to dopamine receptor-blocking agents used to treat psychiatric conditions such as schizophrenia, major depressive disorder, and bipolar disorder [1, 2]. These uncontrolled movements can have profound negative impacts on physical, cognitive, and psychosocial functioning [3].

The Abnormal Involuntary Movement Scale (AIMS), a clinician-reported outcome (ClinRO) measure, is considered the current gold standard for evaluating signs of TD in patients enrolled in clinical trials. The AIMS was originally developed for research purposes to measure the severity (frequency and amplitude) of observed abnormal or uncontrolled movements; however, its adoption for use in routine clinical practice has been limited [2, 4]. In clinical trials, the AIMS is often assessed by several raters following specific procedures. In a clinic setting, clinicians may not have a specific protocol and may measure the severity of TD differently [2]. Moreover, the AIMS score does not measure the functional and social/emotional impact of TD and only measures patient distress with a single item; therefore, it does not adequately capture the full patient burden of TD [2, 4]. In 2 separate advisory panels that provided consensus statements on the assessment of the impact of TD and on use of the AIMS in clinical practice, experts advised that the impact of TD on a patient’s life and functioning should be assessed regularly and that the AIMS may not suffice for understanding the severity and functional impact on patients [2, 3]. A validated, easy-to-use measure for assessing the impact of TD on daily functioning has been recommended to understand the social, physical, vocational, psychological, and psychiatric burden of TD [3]. In 2022, the same advisory panel that provided recommendations on assessing the impact of TD on patients’ lives developed a ClinRO to measure the patient’s experience with TD [3, 5]. The Impact-TD scale considers 4 functional domains: social, psychological/psychiatric, physical, and vocational/educational/recreational [5]. Each domain is scored from 0 (no impact) to 3 (severe impact, significant and detrimental impact) based on clinician observation and patient and caregiver input. This tool has not been validated psychometrically and is not a patient-reported outcome (PRO).

The purpose of this paper is to detail the development and qualitative and quantitative testing of a novel, TD-specific PRO, the Tardive Dyskinesia Impact Scale (TDIS). The methods used in the qualitative research and the psychometric validation of the TDIS are in line with the principles laid out in the Food and Drug Administration (FDA) PRO guidance of 2009 and subsequent patient-focused drug development guidances [6–8].

## Methods

### Background and history related to the TDIS

The TDIS evolved from the Tardive Dyskinesia Rating Scale (TDRS), which was an adaptation of both the Unified Dyskinesia Rating Scale (UDysRS) [9] and the AIMS [10]. The 23-item TDRS was adapted from the UDysRS to measure the severity of dyskinesia, impairment, and disability of the patient. The scale is first completed by the patient/caregiver and then by the clinician. It is similar to the UDysRS and the AIMS in that it attempts to capture the severity of symptoms and signs that are clinically pertinent to TD patients and physicians.

A limitation of the TDRS is that it is not specific to the impact of TD on patients’ lives. Because the TDRS was developed in part from the AIMS and UDysRS, it includes questions about signs of TD as well. These are already captured by the AIMS, which is likely to be used in studies of TD. Moreover, patients were not part of the concept development and item selection of the TDRS, and limited psychometric methods were applied to understand its measurement properties. Since the development of the TDRS, extensive qualitative work (concept elicitation and cognitive debriefing interviews) has been conducted to understand the relevance and interpretation of items and response options. Results of this qualitative work found that patients and caregivers had difficulties understanding the core concepts that were being measured, the instructions for completing the instrument, and the response options of the TDRS [22].

To focus the assessment on the most relevant impacts of TD (e.g. dexterity, mobility, socioemotional impacts), significant modifications to the TDRS were recommended following the results of the qualitative research. In contrast to the development of the TDRS, development of the TDIS was primarily based on patient and caregiver input. Items were selected based on frequently reported impacts, particularly those reported as most bothersome, as well as questions that were free of medical language. In addition, the instructions were simplified, and a reduced set of questions focused on most frequent TD impacts only. A conceptual model of the TDIS was generated as part of this qualitative work to modify and/or replace the TDRS.

### Qualitative research

A literature review and qualitative research were conducted to understand the impact of TD on patients and to confirm the appropriateness of the TDIS as a PRO measure [22, 23]. This effort was accomplished through a targeted PubMed literature review and patient and caregiver interviews in 2 separate qualitative studies; results of the literature review were published in poster form [23]. The objective of the first study was to understand what signs, symptoms, and impacts of TD were relevant from a patient and caregiver perspective, which informed the second study. The second study interviewed patients to determine if the TDIS was interpreted as intended and relevant to patients’ experiences. For both studies, adult patients (aged 18–85 years) were included if they were diagnosed with schizophrenia, schizoaffective disorder, or a mood disorder and with dopamine receptor-blocking agent-induced TD and were aware of abnormal movements (score ≥ 1 [aware, no distress through severe distress] on the AIMS item #10; see Instruments section in Methods for additional details). Additionally for the first study, patients had to have a score of 3 to 4 on the AIMS item #8, and caregivers were included if they were caring for a patient with TD for at least 6 months. For both studies, audio recordings of the interviews were transcribed verbatim, anonymized, and coded and analyzed using Atlas.ti. Patients (and caregivers in the first study) had to provide written informed consent before completing the interviews and were compensated for their time. The studies were approved by the Copernicus Group Independent Review Board.

### Quantitative research

Data from 2 studies (KINECT3 and KINECT4) were used to examine the psychometric properties of the TDIS. KINECT3 was a phase 3, randomized, double-blind, placebo-controlled 52-week trial [21], and KINECT4 was a long-term, open-label 52-week study [24] assessing the safety and efficacy of a VMAT2 inhibitor for the treatment for TD. The main efficacy endpoint in both KINECT3 and KINECT4 was change in total AIMS score from baseline over the study period [21, 24]. The AIMS was scored at Week 6 in KINECT3 and at Week 8 in KINECT4 by 2 central AIMS video raters who were blinded to treatment and time point. Additional efficacy analyses included Patient’s Global Impression of Change (PGIC) and the Clinician’s Global Impression of the Patient’s Change Specific to TD (CGIC-TD). In KINECT3, eligible patients received the VMAT2 inhibitor dosed at 40 mg or 80 mg or placebo for 6 weeks and entered a 42-week extension period, followed by a 4-week washout period [21]. A total of 227 patients (mean age: 56.1 years; 54.2% male) with moderate to severe TD (baseline AIMS score: 10.0) were assessed; approximately 65% of patients had schizophrenia or schizoaffective disorder and 35% had a mood disorder. In KINECT4, patients received the VMAT2 inhibitor dosed at 40 mg or 80 mg (dose escalated at Week 4) for 48 weeks followed by a 4-week washout period [24]. A total of 163 patients (mean age: 57.4 years; 52.8% male) with moderate to severe TD (baseline AIMS score: 10.0) were assessed; 73% of patients had schizophrenia or schizoaffective disorder and 27.0% had a mood disorder. The same analyses were conducted using data from both trials for the purpose of validating results of initial analyses (i.e. showing that the results from both analyses were consistent). Analyses included assessing internal consistency and test–retest reliability, construct validity (factor structure), known-groups validity, and responsiveness to change.

### Instruments

Several different measurements were used in this analysis to evaluate the concurrent validity of the TDIS measurement.

As previously mentioned, AIMS is a ClinRO that is used to evaluate the severity of abnormal or involuntary movements. AIMS contains 12 items, including 3 items (AIMS 8–10) that require global judgment [10]. AIMS items 1–7 assess the severity of abnormal movements in 7 body regions including facial muscles, lips, jaw, tongue, upper and lower extremities, and trunk. Each item is measured on a 5-point scale (0 = none to 4 = severe), with higher scores indicating greater severity.

AIMS item 8 (AIMS 8) assesses overall TD severity, AIMS item 9 (AIMS 9) assesses TD incapacitation, and AIMS item 10 (AIMS 10) assesses patient awareness of TD symptoms and the distress caused by symptoms. Higher scores on AIMS 8 and 9 indicate greater disease severity and incapacitation (0 = none to 4 = severe), and higher scores on AIMS 10 indicate greater disease awareness/distress (0 = no awareness to 4 = aware, severe distress). AIMS item 10 was used in the qualitative research portion of the study to confirm patient awareness of abnormal movement. The single-item PGIC is a PRO that measures the patient’s perception of change related to their condition [28]. The single-item CGIC-TD is a ClinRO that assesses global improvement due to treatment [10]. Both measurements use a rating scale ranging from 0 to 7 as the following: not assessed (0); very much improved (1); much improved (2); minimally improved (3); no change (4); minimally worse (5); much worse (6); very much worse (7). The PGIC and CGIC-TD were used to examine the extent to which patients who showed an improvement in their TDIS total scores also self-identified or were evaluated by the clinician as experiencing a global improvement in their TD symptoms.

### Psychometric validation concepts

#### Internal consistency and test–retest reliability

Internal consistency and test–retest reliability were performed to evaluate how well the items appeared to measure common underlying content and the extent to which the measurement of TD effects was consistent over time. Internal consistency reliability (Cronbach’s alpha) was calculated for the 11 TDIS items for both trials to assess the degree to which the TDIS items captured common underlying content as a total TD impact score. In general, values of coefficient alpha ≥ 0.7 are preferred [25].

Test–retest reliability was calculated for the period between the screening and baseline visits (1–7 days) for both trials. This period was prior to administration of treatment and patients were not expected to experience change in their TD. Calculations of intraclass correlation coefficients were based on Shrout and Fleiss [26].

#### Construct validity

Confirmatory factor analysis (CFA) was performed to evaluate the construct validity of the TDIS by examining its factor structure and gain insights into the degree to which each item loaded on a single factor. The CFA was conducted on the TDIS items for KINECT3 and KINECT4 to assess the hypothetical factor structure that the TDIS is composed of 2 dimensions: 1 set of items that assesses the physical impacts of TD and 1 set that captures the socioemotional impacts of TD. Key goodness-of-fit statistics indicating the fit of the hypothesized model to the actual, observed data included the comparative fit index (values ≥ 0.95 are preferred), the root mean square error of approximation (values ≤ 0.08 are preferred), and the standardized root mean square residual (values < 0.06 are preferred) [27].

Longitudinal construct validity was assessed by correlating change from baseline TDIS with change from baseline AIMS and the CGIC-TD and the PGIC. This included examining to what extent the patterns of TDIS scores are logically based on the response anchors of the PGIC and the CGIC-TD.

#### Known-groups validity

A series of analyses were performed to evaluate the known-groups validity of the TDIS. Known-groups validity was assessed 3 ways: (1) correlating the AIMS and TDIS; (2) comparing mean TDIS total scores for each level of CGIC-TD and PGIC; and (3) assessing mean TDIS total scores for each level of severity of the patient’s most bothersome movement at baseline.

Mean TDIS total scores for each level of severity of the patient’s most bothersome movement at baseline were examined. Each person who completed the TDIS was also asked, “Thinking about all of your uncontrollable movements, which one bothers you the most (e.g. facial muscle movement, lip, or mouth movement, etc.)?” A follow-up question then asked how bothersome the movement is to them on a 11-point scale from 0 (not at all bothersome) to 10 (extremely bothersome).

#### Responsiveness to change

To understand the responsiveness to change in TDIS, the extent of the change in the TDIS score from baseline to later visits in each trial and whether the change followed a similar pattern to that of the clinician-assessed AIMS was evaluated. Change was assessed from baseline to Week 52, which included a drug-free washout period from Weeks 48 to 52 in both KINECT3 and KINECT4.

All analyses were conducted using SAS Version 9.4 (Cary, NC) or Mplus Version 8.4 (Los Angeles, CA).

## Results

### Qualitative research

Twenty-two patients with TD and 11 caregivers from 5 clinical sites in the United States were interviewed to elicit the main signs, symptoms, and impacts of TD (concept elicitation) and subsequently interviewed to assess distinct parts of the TDRS (cognitive debriefing) in the first study. Cardinal signs, symptoms, and impacts achieved saturation, indicating that a sufficient number of interviews were conducted to inform the construction of the PRO. The most common movements reported were in the tongue (60.6%) and jaw (57.6%), which were also reported as the most bothersome movements [22]. The most common impacts of TD were unwanted social attention (75.8%), difficulty speaking (63.6%), and social isolation (54.5%). Concepts endorsed in the TDRS by more than half of the participants included difficulty chewing and swallowing, dressing, handwriting, participating in hobbies, hygiene, dyskinesia pain, difficulty in public/social settings, speech, and walking and balance. The concepts reported and endorsed in the concept elicitation and cognitive debriefing portion of the interviews helped inform the development of the TDIS items (Table 1).

**Table 1:** TDIS domains, TDIS items, and mapping to TDRS items

Domain	TDIS item	Corresponding TDRS item
Mouth/throat function	1. Speech	Item 2. Speech
	2. Mouth noises	Not applicable
	3. Swallowing	Item 3. Chewing and swallowing
Dexterity	4. Gripping	Item 4. Eating tasks Item 5. Dressing Item 6. Hygiene Item 8. Hobbies
	5. Writing	Item 7. Handwriting
Mobility	6. Walking 7. Balance	Item 9. Walking and balance
Pain	8. Leg pain	Item 12. Dyskinesia pain
Social	9. Unwanted attention	Item 10. Public and social settings
Emotional	10. Embarrassed 11. Self-conscious	Not applicable

*TDIS* Tardive Dyskinesia Impact Scale, *TDRS* Tardive Dyskinesia Rating Scale

**Fig. 1:**
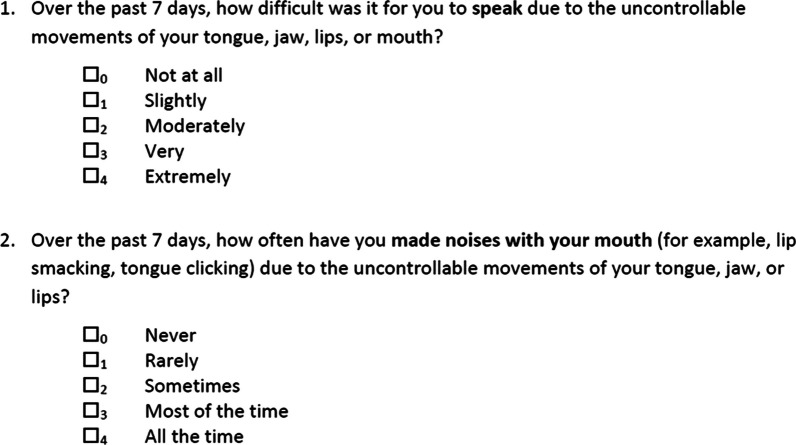
Sample of TDIS questionnaire. *TDIS* Tardive Dyskinesia Impact Scale

In the second study, which focused on cognitive debriefing of the TDIS with 20 patients from 4 US-based clinical sites, most patients (*n* = 18, 90.0%) interpreted the instructions as intended and indicated that the instructions were clear. The word “dyskinesias” in the instructions was considered difficult by 7 of the participants and as a result was changed to “uncontrollable movements.” Most patients (70%) could recall their experiences within the stated time period (over the past 7 days). Some patients (35%) reported it was difficult to think about the “past 7 days”; 3 patients suggested using a shorter time period. However, as most patients understood the intended recall period, no change was made. Participants were also asked which TDIS concepts they considered most relevant to their experience with TD. The concepts most highly endorsed by patients were receiving unwanted attention (*n* = 15, 75.0%), difficulty speaking (*n* = 14, 70.0%), and feeling embarrassed (*n* = 14, 70.0%). The most common issue was ensuring patients would focus on the core concept in each item and therefore bolded text was used to indicate the main concept of interest in each item; the most consistently challenging concept was self-consciousness, which is difficult to interpret across all populations. Because the concept of self-consciousness was considered highly relevant based on feedback from the concept elicitation of the TDRS, no changes were made to the question besides bolding the key concept. Based on the patient feedback, minor changes were made to the TDIS questions, including bolding the key concept in each question (Fig. 1), adding typing to item 5 (writing), including instructions for item 6 (walking), and generalizing pain (item 8) so it was not specific to legs or lower body.

The final TDIS is an 11-item questionnaire developed to understand how TD affects current daily functioning over the previous 7 days. Each question is scored on a 5-point Likert scale, ranging from 0 (no impact) to 4 (most impact). TDIS total score can range from 0 to 44, with higher scores representing greater TD impact. A sample of the TDIS questionnaire is shown in Fig. 1.

### Quantitative research

#### Internal consistency and test–retest reliability

The alpha reliabilities were 0.88 for KINECT3 and 0.90 for KINECT4, indicating that these 11 items reliably measure common underlying content. In addition, none of the items were found to be problematic and thus did not need to be deleted to improve the overall alpha. The alpha-if-deleted ranged from 0.87 to 0.88 for KINECT3 and 0.89 to 0.90 for KINECT4. The intraclass correlation coefficients for both trials was 0.83, reflecting good test–retest reliability.

#### Construct validity: confirmatory factor analysis

**Fig. 2:**
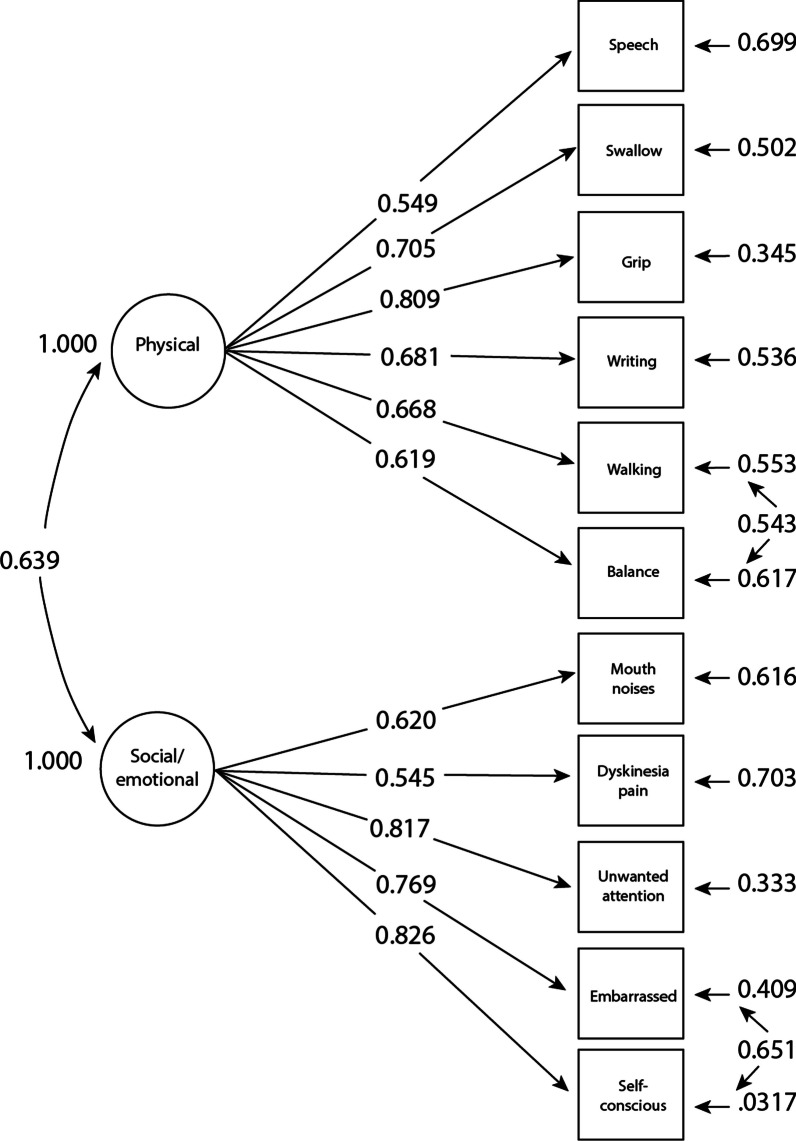
KINECT3 CFA of the 11-Item TDIS. *CFA* Confirmatory factor analysis, *TDIS* Tardive Dyskinesia Impact Scale

The CFA was nearly identical for KINECT3 and KINECT4. While there appeared to be good support for a 2-factor solution to the underlying content of the TDIS, there was also evidence of a second-order factor (Fig. 2). The large correlation between the physical and the socioemotional factors in both trials suggested that one possible explanation accounting for this strong correlation was that the variability in each of the 2 factors was the result of another, higher-order factor. The goodness-of-fit results for KINECT3 were as follows: comparative fit index, 0.93; root mean square error of approximation, 0.084 (90% CI 0.07–0.10); and standardized root mean square residual, 0.069, which were similar for KINECT4. We obtained factor loadings for the paths between the second-order factor and the physical and socioemotional factors. For KINECT3, those loadings were 0.88 to physical and 0.72 to socioemotional. For KINECT4, the respective loadings were 0.82 and 0.91. This second-order factor would represent the total TD impact experienced by the patient.

The change from baseline in the AIMS and the TDIS showed weak correlations in KINECT3 but more mixed strengths of correlations in KINECT4 (Table 2). In KINECT3, the change from baseline in the AIMS was more strongly correlated with the CGIC-TD, while the change from baseline in the TDIS was more strongly correlated with the PGIC. In KINECT4, the change from baseline in the AIMS was more strongly associated with the CGIC-TD vs PGIC (0.65 vs 0.37), while change from baseline in the TDIS had a slightly stronger correlation with the CGIC-TD compared with that of the PGIC (0.34 vs 0.30). This may be due to differences in study designs of the trials (as KINECT3 was a randomized, double-blinded, placebo-controlled trial and KINECT4 was an open-label trial) and the time difference between the primary endpoint in each trial (KINECT3 was 6 weeks and KINECT4 was 8 weeks).

**Table 2:** Correlations between change in AIMS and TDIS to CGIC-TD and PGIC in KINECT3 and KINECT4

	KINECT3 Week 6^a^ (*n* = 201)			KINECT4 Week 8 (*n* = 148)		
	CGIC-TD	PGIC	ΔAIMS	CGIC-TD	PGIC	ΔAIMS
ΔAIMS	0.34	0.13	–	0.65	0.37	–
ΔTDIS	0.24	0.30	0.14	0.34	0.30	0.26

ΔAIMS Change from baseline to end of double-blind period in AIMS, Δ*TDIS* Change from baseline to end of double-blind period in TDIS, *AIMS* Abnormal Involuntary Movement Scale, *CGI-TD* Clinician Global Impression of Change – Tardive Dyskinesia, *PGIC* Patient Global Impression of Change, *TDIS* Tardive Dyskinesia Impact Scale

^a^Refers to the end of the double-blind, placebo-controlled period

#### Known-groups validity

The correlation between AIMS and TDIS in KINECT3 at baseline was 0.26 and 0.28 at week 6 (end of the double-blind, placebo-controlled period). For KINECT4, the correlation between AIMS and TDIS at baseline was 0.31 and 0.21 at Week 8, which roughly corresponded to the end of the double-blind period in KINECT3 [29]. The correlations between the AIMS and the TDIS were weak in both trials, indicating that there is little overlap in content between the 2 measures.

Mean TDIS total scores were compared with each level of PGIC and CGIC-TD and change from baseline in TDIS total scores for each level of PGIC (Table 3). There was a significant difference in TDIS total score by PGIC score overall (*P* = 0.002); however, there was only a significant difference between PGIC score of 1 compared with scores 2–5 when TDIS scores were compared across individual PGIC scores. The results from KINECT3 and KINECT4 generally showed that for patients with an impression of improvement in their condition (PGIC score 1–3), their TDIS total scores were lower (i.e. less TD impact) than those who rated the change in their condition as worse. A similar pattern existed when examining change from baseline in TDIS total scores; those who rated their condition as improved had greater improvements in their TDIS total scores. Results were similar, although not as defined, when examining mean TDIS total scores and change from baseline in TDIS total scores relative to the CGIC-TD.

**Table 3:** Breakdown of mean TDIS total score by each PGIC score: End of double-blind KINECT3

PGIC score	Number of patients	Total TDIS score, mean (SD)	Change from baseline to Week 6 in TDIS total score, mean (SD)
1	22	3.5 (4.31)	− 7.68 (8.60)
2	44	11.09 (8.31)	− 5.77 (7.04)
3	83	12.36 (8.63)	− 3.90 (6.19)
4	46	12.13 (8.53)	− 1.50 (5.78)
5	6	15.0 (6.57)	0.67 (6.77)
*Total*	201	11.14 (8.52)	− 4.04 (6.86)

*PGIC* Patient Global Impression of Change, *SD* Standard deviation, *TDIS* Tardive Dyskinesia Impact Scale

PGIC rated on the following scale: 1 = very much improved, 2 = much improved, 3 = minimally improved, 4 = not changed, 5 = minimally worse, 6 = much worse, 7 = very much worse

No patient reported a score of 6 (much worse) or 7 (very much worse)

Mean TDIS total scores were compared with each level of severity of the patient’s most bothersome movement at baseline. These results show that as a movement became more bothersome, TD impact worsened (i.e. greater TD impact). The baseline correlation between TDIS total score and how bothersome their most bothersome movement was 0.56 for KINECT3 and 0.57 for KINECT4.

### Responsiveness to change

**Fig. 3:**
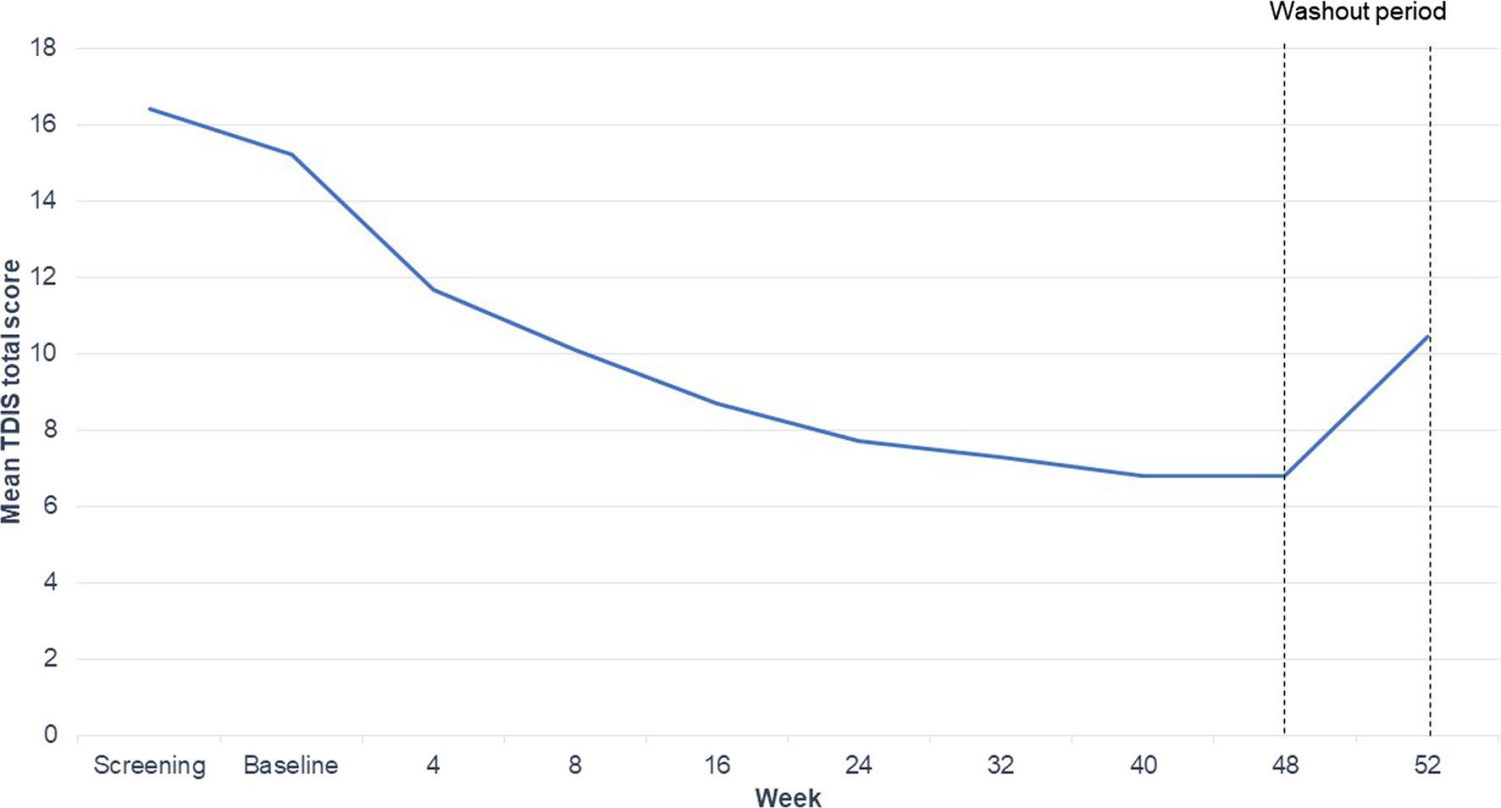
KINECT3 mean TDIS total score through Week 52. *N* = 233 at baseline to 129 at Week 52. *TDIS* Tardive Dyskinesia Impact Scale

**Fig. 4:**
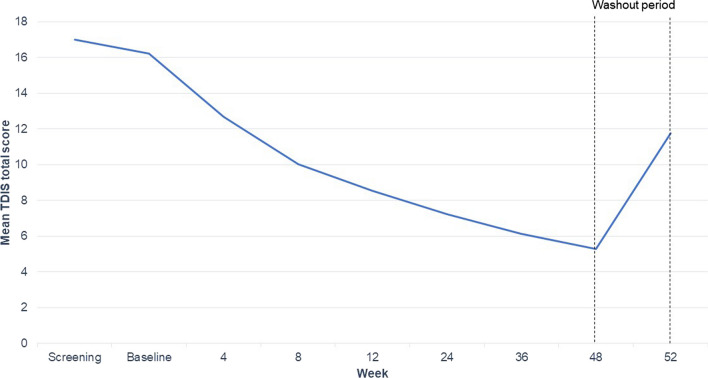
KINECT4 mean TDIS total score through Week 52. *N* = 167 at baseline to 103 at Week 52. *TDIS* Tardive Dyskinesia Impact Scale

Mean TDIS total scores were plotted for the main patient visits from baseline to Week 52 for KINECT3 (Fig. 3) and KINECT4 (Fig. 4). On average, baseline TDIS total scores were only about one-third (16–17 points) of the maximum score possible (44 points) for both trials, indicating that at baseline, TD has a moderate impact on patients. By Week 48, mean scores were 6 to 7 points, showing that TD impact had improved to have little impact, on average. During the washout period in both trials, TDIS scores worsened, showing that the TDIS is responsive to change in treatment.

## Discussion

Results from qualitative and quantitative research indicated that the TDIS is a valid and reliable measure of the impact of TD on patients. The TDIS evolved from the TDRS, which was an adaptation of the UDysRS and the AIMS. Thus, the TDIS has a good pedigree for capturing the severity of effects of TD from a patient’s perspective. Patient and caregiver interviews confirmed that the TDIS appropriately captured key patient experiences of the physical and socioemotional impacts of TD. In addition, most patients interpreted TDIS items as intended, and more than half of participants reported experiencing the concept measured in most items (e.g., speech, walking, leg pain; see Table 1 for all TDIS items).

Using data from KINECT3 and KINECT4 trials, results of numerous psychometric analyses, CFA, known-groups validity, internal consistency, test–retest reliability, and responsiveness to change consistently reflected robust psychometric qualities of the TDIS. Results of the CFA showed support for a 2-factor solution of the 11-item TDIS, with 1 factor capturing the physical impacts of TD and 1 factor representing the socioemotional effects of TD. In addition, there was empirical support for a second-order factor representing the overall TD impact experienced by patients. The results of the CFA suggested that researchers could examine patient responses on the TDIS either as a total score (based on the second-order factor) or on the individual first-order factors. This characteristic of the TDIS enables an assessment of the influence of different variables on the physical or socioemotional components of a patient’s TD experience or the consequences of these 2 components of the TDIS on other outcomes, such as depression. A separate manuscript is currently under way to better understand these results.

The correlations between the AIMS and the TDIS were weak in both trials, indicating that there is little overlap in content between the 2 measures. These 2 instruments are designed to measure different aspects of TD. The AIMS is designed to allow the clinician to assess the severity of signs of TD, while the TDIS is designed to allow the patient to report the difficulty and frequency of impacts of TD. The recently developed ClinRO instrument, the IMPACT-TD, was similarly designed to assist clinicians in measuring the functional impact of TD [5]. However, it was developed by a panel of clinicians and did not include patients or caregivers as part of the development process. As noted in the patient-focused drug development guidance 3, patient experience data should provide information about a patient’s experience with the disease, including the physical and psychosocial impact [8]. Additionally, the guidance suggests the status of a patient’s disease or health condition should come directly from the patient without influence from others, including clinicians [8]. In this manner, the TDIS can help fill a gap in understanding of TD from the patient perspective, as there is no other validated specific measure available to understand how TD affects patients’ physical and socioemotional quality of life.

The TDIS showed change over 48 weeks of treatment and 4 weeks of washout in the 2 trials, indicating that it is responsive to change. It is important to note the consistency of this change across 2 differently designed trials. Moreover, as time-on-treatment continued, TD impact decreased from the patient’s perspective, suggesting that the TDIS could be useful as a monitoring tool to evaluate changes in the patient’s TD impact resulting from treatment.

Some limitations exist for these analyses. First, no clinically important change value or responder definition was calculated since this current study was focused on the main measurement properties of the TDIS. The values associated with meaningful change or a responder definition are planned for a separate publication. Second, the current study does not evaluate the TDIS as an efficacy measure, and the clinical trials were not designed or powered to detect treatments effects on the TDIS. Results from the exploratory analysis of KINECT3 showed improvement in the TDIS from baseline to Weeks 2 and 4, but the changes were comparable for all 3 arms (i.e. 40 mg and 80 mg of VMAT2 inhibitor and placebo). Further examination of this is warranted. Lastly, TD is a heterogenous condition, and not all concepts included in the TDIS may be applicable for all patients and not all impacts reported by patients are included in the TDIS. The TDIS provides a starting point for understanding the impact of TD on the patient’s everyday life, which can be used along with clinician assessment and discussion of symptoms between the patient, clinician, and caregiver.

## Conclusion

Scores from the AIMS represent a clinician’s assessment of the severity (frequency and amplitude) of observed abnormal or uncontrolled movements and provide little information from the patient’s perspective about the impacts of those uncontrolled movements in daily functioning. The results presented from these analyses indicate that the AIMS and the TDIS appear to capture different content and are differentially related to patient and clinician assessments of improvement in TD. The TDIS, as a PRO when implemented in practice, should complement the AIMS, as the measures together can provide a comprehensive picture of TD severity, signs, and impacts on TD patients.

The results from these analyses may be helpful in designing future studies by evaluating a patient’s perspective on the impact of TD and observing how the TDIS relates to the PGIC, the CGIC-TD, AIMS total score, and other PROs. The TDIS can be easily administered in a clinician’s office or at a patient’s home to provide insight about TD impact and whether the impact is lessening or increasing. Used together with ClinROs (e.g. AIMS, IMPACT-TD) and other PROs (e.g. quality of life measures), the TDIS could help clinicians gain important insights into patients’ daily experiences with TD and provide a more holistic assessment of this disorder.

## Data Availability

The datasets analyzed during the current study are not publicly available due to patient confidentiality.

## Abbreviations

ΔAIMS: Change from baseline to end of double-blind period in AIMS
ΔTDIS: Change from baseline to end of double-blind period in TDIS
AIMS: Abnormal Involuntary Movement Scale
CFA: Confirmatory factor analysis
CGIC-TD: Clinician’s Global Impression of the Patient’s Change Specific to Tardive Dyskinesia
ClinRO: Clinician-reported outcome
FDA: Food and Drug Administration
PGIC: Patient Global Impression of Change
PRO: Patient-reported outcome
TD: Tardive dyskinesia
TDIS: Tardive Dyskinesia Impact Scale
TDRS: Tardive Dyskinesia Rating Scale
UDysRS: Unified Dyskinesia Rating Scale
